# 9.4 MHz A-line rate optical coherence tomography at 1300 nm using a wavelength-swept laser based on stretched-pulse active mode-locking

**DOI:** 10.1038/s41598-020-66322-0

**Published:** 2020-06-09

**Authors:** Tae Shik Kim, JongYoon Joo, Inho Shin, Paul Shin, Woo Jae Kang, Benjamin J. Vakoc, Wang-Yuhl Oh

**Affiliations:** 1 Department of Mechanical Engineering, KAIST, Daejeon, Republic of Korea; 2 KI for Health Science and Technology, KAIST, Daejeon, Republic of Korea; 3 Wellman Center for Photomedicine, Massachusetts General Hospital and Harvard Medical School, Boston, MA USA

**Keywords:** Imaging and sensing, Biophotonics

## Abstract

In optical coherence tomography (OCT), high-speed systems based at 1300 nm are among the most broadly used. Here, we present 9.4 MHz A-line rate OCT system at 1300 nm. A wavelength-swept laser based on stretched-pulse active mode locking (SPML) provides a continuous and linear-in-wavenumber sweep from 1240 nm to 1340 nm, and the OCT system using this light source provides a sensitivity of 98 dB and a single-sided 6-dB roll-off depth of 2.5 mm. We present new capabilities of the 9.4 MHz SPML-OCT system in three microscopy applications. First, we demonstrate high quality OCTA imaging at a rate of 1.3 volumes/s. Second, by utilizing its inherent phase stable characteristics, we present wide dynamic range *en face* Doppler OCT imaging with multiple time intervals ranging from 0.25 ms to 2.0 ms at a rate of 0.53 volumes/s. Third, we demonstrate video-rate 4D microscopic imaging of a beating *Xenopus* embryo heart at a rate of 30 volumes/s. This high-speed and high-performance OCT system centered at 1300 nm suggests that it can be one of the most promising high-speed OCT platforms enabling a wide range of new scientific research, industrial, and clinical applications at speeds of 10 MHz.

## Introduction

Despite increasing 1000-fold in imaging speed since its initial demonstration, there are many applications of optical coherence tomography (OCT) that would benefit from the systems that are faster than what is broadly available. Imaging speeds of the OCT systems depend on the capabilities and limitations of the technologies upon which the systems are based. This has been a defining characteristic of OCT, and it has created fertile ground for innovation. The result has been new architectures supporting high-speed imaging^1–6^, new detection systems for accelerating the capture of imaging signals^1,2,5–7^, and new high-speed optical source designs^3,4,8–19^. The resulting speed gains have led to many of the most impactful translations of OCT. Examples include wide-field imaging of large organs^20–23^, angiographic imaging based on extensive oversampling^24–33^, and rapid volumetric imaging of dynamic events^33–38^.

Over the past decade, a substantial effort was directed toward increasing the repetition rate of wavelength-swept sources used in OCT. Most of the resulting source technologies are based on a rapidly-tuned mechanical optical filter, e.g., a MEMS mirror or Fabry-Perot etalon, and provide intrinsic repetition rates up to approximately several hundred kilohertz. To further increase speed, optical delay line buffering has been implemented. In this approach, the laser output is optically copied, delayed, and interleaved. Optical buffering is most effective when used to increase laser speeds by small factors, e.g., 2-times or 4-times buffering. For larger factors, the complexity and cost of the buffering system becomes significant. As a result, mechanical sources in combination with optical buffering have raised OCT system speeds to the level of several megahertz, but may not be the right strategy for 10 MHz and faster sources.

To create faster sources, many research teams have focused on non-mechanical (or akinetic) laser architectures. To date, a number of non-mechanical source designs have been proposed^39–47^. Of these, sources that rely on chromatic dispersion have provided some of the highest operating speeds. Stretched-pulse active mode locking (SPML) architectures are an example of this category and are based on intracavity inclusion of positive and negative dispersion. SPML sources have been demonstrated based on the use of intra-cavity fiber spools^44^, and intracavity chirped fiber Bragg gratings (CFBGs)^45,46^. In the later, a single CFBG several meters in length replaces tens of kilometers of fiber. However, to date, only long-length fiber-based SPML lasers have been used to perform OCT imaging^47,48^.

In this work, we demonstrate 9.4 MHz A-line rate OCT imaging based on the CFBG-SPML laser. This is the first demonstration of an SPML laser and an OCT system at 1300 nm, which has been the preferred operating wavelength for non-retinal biomedical OCT applications (prior SPML lasers and systems operated at 1550 nm^44,46–48^), and the first to perform OCT imaging using a CFBG-SPML laser. The SPML source provided a 100 nm (1240–1340 nm) optical bandwidth and generated a linear-in-wavenumber sweep that does not require resampling or k-clocked acquisition. The intrinsic speed of the source was 4.7 MHz, and 2x optical buffering was used to reach 9.4 MHz. The sensitivity of the system was measured to be 98 dB. The single-sided 6-dB coherence length of the laser was 2.5 mm. Using this system, we acquired high-quality OCT angiography (OCTA) images (748 A-lines × 750 B-scans) that visualize microvasculature in the rodent brain at a rate of 1.3 volumes per second. Wide dynamic range Doppler OCT imaging of rodent brain (748 A-lines × 750 B-scans) was presented at a rate of 0.53 volumes per second by acquiring 3D Doppler OCT data with eight different time intervals ranging from 0.25 ms to 2 ms. We also demonstrated video-rate volumetric imaging of a beating *Xenopus* embryo heart (360 A-lines × 360 B-scans) at a rate of 30 volumes per second. These results suggest CFBG-SPML sources may be an enabling technology for high-quality and multi-functional OCT imaging at speeds of 10 MHz.

## Results

### 1300 nm SPML wavelength-swept laser

**Figure 1:**
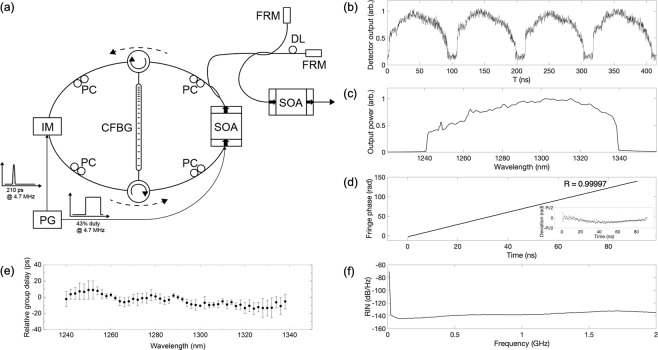
SPML laser design and performance. (**a**) Schematic of the SPML laser at 1300 nm. IM, intensity modulator; PG, pulse generator; PC, Polarization controller; CFBG, chirped fiber Bragg grating; SOA, semiconductor optical amplifier; FRM, Faraday rotating mirror; DL, delay line. (**b**) Laser trace. (**c**) Optical spectrum. (**d**) Unwrapped fringe phase measured with a partial reflector in the sample arm (Inset: Deviation of fringe phase from a linear fit). (**e**) Relative group delay over a single cavity roundtrip. (**f**) Measured RIN.

Figure 1(a) shows a schematic of the 1300 nm SPML wavelength-swept laser operating at a 9.4 MHz repetition rate. It is an actively mode-locked laser with a semiconductor optical amplifier (SOA, Thorlabs BOA1130S) and an electro-optic intensity modulator (EOM, iXblue Photonics MX1300-LN-10). An electrical pulse of ~210 ps width from a bit pattern generator (Sympuls PAT 5000, max data rate: 5Gbit/s) that repeats in resonance with the cavity round trip time (213 ns = (4.7 MHz)^−1^) was supplied to the intensity modulator through a driver module (iXblue Photonics DR-PL-20-MO) to modulate the optical transmission of the laser cavity for active mode-locking. A customized 10-meter-long CFBG (Proximion) provided linear-in-wavenumber group delay (±930 ps/nm average dispersion) across a 100 nm wavelength range from 1240 nm to 1340 nm. The CFBG was located between a pair of circulators in the theta-cavity configuration^45,46^. The EOM output pulse was stretched by reflecting from the CFBG to produce a wavelength-swept output. After the output coupler, the wavelength-swept light remaining in the cavity was directed to the opposite side of the same CFBG to compress the light back to a short pulse. To suppress unwanted lasing of light that passes through the CFBG, the SOA gain medium was modulated on/off with a duty cycle of 44% in every cavity round trip in such a way that the SOA was turned off when the unwanted light returned to the SOA. The swept-laser output was taken from a 10% tap coupler, and the repetition rate and duty cycle of this output was doubled using a 2x optical buffer. This doubled output was amplified using a booster SOA, resulting in 9.4 MHz wavelength sweep (88% duty cycle) with an average output power of 80 mW.

Figure 1(b,c) show the laser output trace in time and the integrated output spectrum, respectively, that confirm a 100 nm sweep from 1240 nm to 1340 nm at a repetition rate of 9.4 MHz. Unwrapped phase of the fringe signal measured with a partial reflector in the sample arm (Fig. 1(d)) confirms the linear-in-wavenumber sweep of the laser. The wavelength-dependent relative group delay for a cavity round trip was measured using the modulation phase-shift method^49^ as shown in Fig. 1(e). The variation in group delay across 100 nm was smaller than 30 ps. Note that the group delay variation is significantly smaller than the mode-locked pulse width of 130 ps (FWHM) that was measured right after the intensity modulator using a high-speed receiver (New Focus 1474-A, 3-dB bandwidth: 35 GHz) and a sampling oscilloscope (Pico Technology 9301–25, 3-dB bandwidth: 25 GHz). For the pulse width measurement, we temporarily connected a 1% tap coupler after the intensity modulator.

The point spread functions (PSFs) measured at different depths using the 35 GHz high-speed receiver and the 25 GHz sampling oscilloscope show that the laser provided a single-sided 6-dB coherence length of 2.5 mm. The coherence length of the laser is determined by the instantaneous linewidth of the wavelength-swept laser, and the instantaneous linewidth, δλ, can be approximated as

$$\delta \lambda =\frac{\delta t}{D},$$

where D is the dispersion of the CFBG and δt is the temporal width of the optical pulse exiting the EOM. Using δt = 130 ps and D = 930 ps/nm, an instantaneous linewidth of δλ = 0.14 nm is predicted by Eq. 1, which corresponds to a single-sided 6-dB coherence length of 2.6 mm, close to the measured value of 2.5 mm.

The relative intensity noise (RIN) of the laser was measured using the 35 GHz high-speed receiver and a high-speed digitizer (Alazartech ATS9373, 12 bit, 4 GS/s). RIN was calculated after subtracting the background time-trace of the laser from each A-line. The background time-trace was calculated by averaging several hundred A-line sweeps^50^. This is analogous to background subtraction that is commonly performed in OCT imaging. RIN levels were less than than −137 dB/Hz over a frequency range up to 2 GHz (Fig. 1(f)).

### OCT system

**Figure 2:**
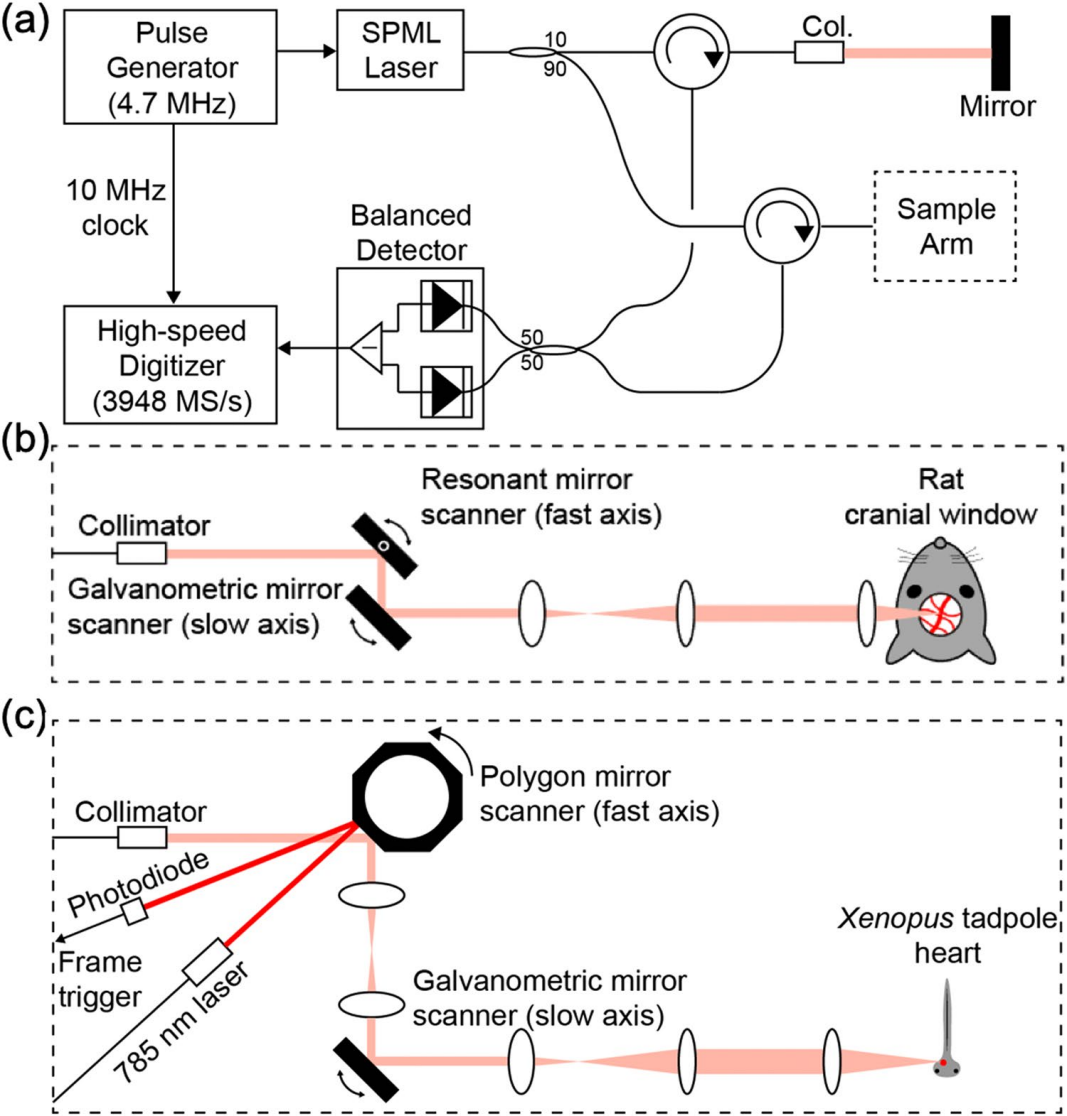
OCT system configuration. (**a**) Configuration of the OCT system. (**b**) A schematic of the sample arm optics for OCTA and Doppler OCT imaging of a rat brain. (**c**) A schematic of the sample arm optics for video-rate 3D OCT imaging of a beating *Xenopus* embryo heart.

The 9.4 MHz A-line rate OCT system using the SPML laser is shown in Fig. 2(a). The interference signals were detected by a balanced receiver (Thorlabs PDB480C-AC, 3-dB bandwidth: 1.6 GHz) and digitized by a 4 GS/s high-speed digitizer (Alazartech ATS9373). A 10 MHz reference clock from the bit pattern generator (electric pulse generator) phase-locked the digitizer. The digitizer acquired the OCT signal at a sampling rate of 3,948 MHz providing 420 samples per A-line. The sensitivity of the system was measured to be 98 dB with a power of 45 mW on the sample^4^.

To efficiently utilize the high-speed imaging capability, we used a 4-kHz resonant scanning mirror (EOPC SC-30), which was also phase-locked to the 10 MHz reference clock from the bit pattern generator, for the fast-axis beam scan as shown in Fig. 2(b). 2,350 A-lines (forward and backward scans) were acquired per B-scan, which resulted in 748 effective A-lines per B-scan after scan-linearization. A regular galvanometric scanning mirror was used for the slow-axis beam scan.

For OCTA imaging, a stepwise sawtooth scan pattern was used for the slow-axis beam scanning (see Supplement 1). A total of twenty-five steps with a spacing corresponding to thirty B-scans constituted a volume scan. Four sawtooth scans were repeated in each step, and thirty-one B-scans were acquired at different slow-axis locations during each sawtooth scan. Three OCTA volume data, each consisting of 748 A-lines × 750 B-scans, were acquired from the four sets of repeated sawtooth scans with a 7.75 ms time interval and averaged to provide a noise-reduced 3D OCTA image at a rate of 1.3 volumes/s. The transverse and axial resolutions were 9.4 μm and 9.6 μm in the air, respectively.

Since the SPML wavelength-swept laser was inherently phase-stable, and the light source, digitizer, and beam scanning unit were all phase-locked, Doppler OCT imaging was achieved without the need for additional phase stabilization or phase calibration^47^. For Doppler OCT imaging, a stepwise scan pattern was used for the slow-axis beam scanning (see Supplement 1). Ten B-scans at the same slow-axis location were acquired, and the imaging beam was subsequently stepped to the next location. Eight 3D Doppler OCT data sets generated with eight different time intervals ranging from 0.25 ms to 2 ms enabled wide dynamic range Doppler OCT imaging (748 A-lines × 750 B-scans) at a rate of 0.53 volumes/s.

For the video-rate volumetric OCT imaging, the fast-axis beam scan used a polygon mirror scanner as shown in Fig. 2(c). A polygon mirror with 36 facets (Lincoln laser SA34/DT-36–250–025) was rotated at 300 rps, providing a B-scan rate of 10.8 kHz. We acquired images only when the imaging beam was not clipped at the edges of the polygon facets, which approximately resulted in a 42% duty cycle and 360 A-lines per B-scan. We acquired a 3D dataset comprising 360 A-lines × 360 B-scans at a rate of 30 volumes/s. Unlike the imaging with a resonant scanning mirror that was phase-locked with the data acquisition, there existed a non-negligible drift in beam scanning speed when using the polygon mirror scanner. As such, a collimated beam at 780 nm wavelength (Thorlabs CPS780S) was reflected from the polygon mirror and detected with a silicon photodiode (Hamamatsu S1223-01). This signal was used to generate a reference position for the start of each frame. The transverse resolution in this configuration was 6.3 μm in the air.

### High-quality OCTA

**Figure 3:**
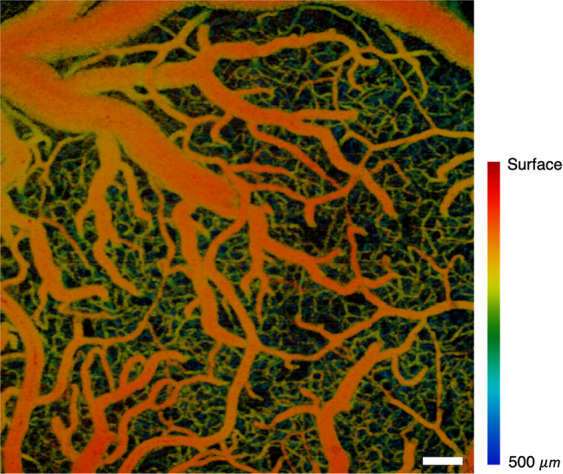
A depth-projected *en face* OCTA image of a rat brain with depth encoded in color acquired at 1.3 OCTA volumes per second. Scale bar: 250 μm.

Figure 3 shows the depth-projected *en face* OCTA image over a field of 3 mm × 3 mm (748 A-lines × 750 B-scans) in the cranial window implanted on a rat cortex. Four 3D OCTA data sets were acquired in 769 ms (1.3 volumes/s) using the stepwise sawtooth scan pattern for the slow-axis scan, which were averaged to reduce noise. The volumetric projection^51^ of the 3D microvasculature down to 500 μm depth from the tissue surface was presented with depth encoded by color.

### Wide dynamic range Doppler OCT

**Figure 4:**
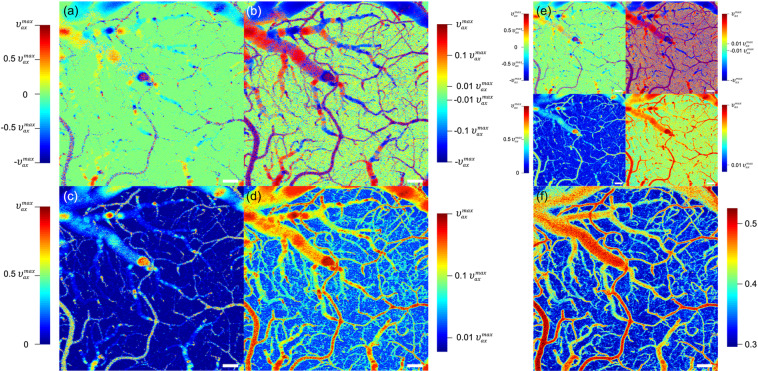
Wide dynamic range Doppler OCT imaging of a rat brain. *En face* projections of the axial blood flow speed in (**a**) linear scale and (**b**) logarithmic scale. *En face* projections of the magnitude of the axial blood flow speed in (**c**) linear scale and (**d**) logarithmic scale. (**e**) *En face* projections of the axial blood flow speed and the magnitude of the axial blood flow speed obtained with a single time interval of 0.25 ms. (**f**) An *en face* projection of the mean complex decorrelation obtained with the same six time intervals used in the wide dynamic range Doppler OCT. Scale bar: 250 μm.

The same region was imaged using the stepwise slow-axis scan at a rate of 0.53 volumes/s (1.88 s/volume) for wide dynamic range Doppler OCT. Of the Doppler OCT data acquired using the eight different time intervals ranging from 0.25 ms to 2 ms, we utilized six depth-projected (maximum projection) Doppler phase difference data acquired with 0.25 ms, 0.50 ms, 1.0 ms, 1.5 ms, and 2.0 ms. The wide dynamic range Doppler phase difference, Φ, was determined by selectively using the Doppler phase difference obtained with each time interval as described in supplementary materials. Figure 4 shows the *en face* image of the axial blood flow speed obtained from Φ. The fastest measurable axial blood flow speed was $\pm {\upsilon }_{ax}^{max}$ = ±0.96 mm/s that corresponds to the Doppler phase difference of ±π with the shortest time interval of 0.25 ms, assuming tissue refractive index of 1.35. Figure 4(a,b) show the axial blood flow speed in linear scale and logarithmic scale, respectively. Figure 4(c,d) show the magnitude (absolute value) of the axial blood flow speed in linear scale and logarithmic scale, respectively. The axial blood flow speed images acquired with multiple time intervals [Fig. 4(a–d)] show a significantly wider dynamic range compared with those obtained using a single time interval of 0.25 ms [Fig. 4(e)]. It is worth noting that the image of the magnitude of the axial blood flow speed presented in logarithmic scale [Fig. 4(d)] appears similar to the quantitative OCTA image generated using the mean complex decorrelation (mCD), which showed a linear fit to a logarithmic function of the blood flow speed^52^, with the same six time intervals [Fig. 4(f)].

### Video rate 4D imaging of a beating *Xenopus* embryo heart

**Figure 5:**
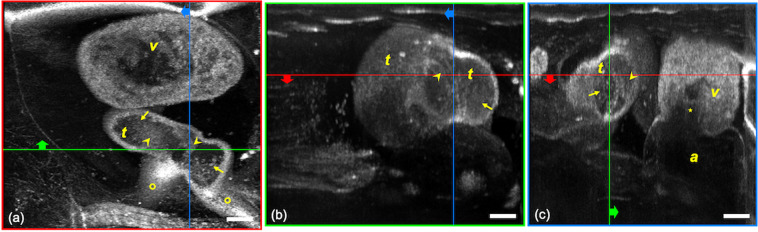
Video-rate 3D OCT imaging of a beating *Xenopus* embryo heart. 3D volume rendered (**a**) coronal, (**b**) axial, and (**c**) sagittal views of the embryo heart. v, ventricle; t, truncus arteriosus; a, atrium; arrows, blood cells; arrow heads, spiral valves; circles, aortic arches; star, atrioventricular valve. Scale bar: 100 μm. Real time videos are provided in Supplement.

Figure 5 shows the video-rate 3D OCT imaging of a beating *Xenopus* embryo heart. A 3D volume corresponding to 360 A-lines × 360 B-scans (1 mm × 1 mm) was imaged at a rate of 30 volumes/s. Figure 5(a) shows the 3D volume-rendered (ImageJ, US National Institutes of Health) coronal view of the embryo heart below the planes marked by red lines in Fig. 5(b,c). Figure 5(b) shows the axial view above the plane marked by a green line in Fig. 5(a) and to the right side of the plane marked by a green line in Fig. 5(c). Figure 5(c) shows the sagittal view to the left side of the planes marked by blue lines in Fig. 5(a,b). Fine 3D internal structures of the embryo heart such as the ventricle, truncus arteriosus, atrium, spiral valve, atrioventricular valve, and aortic arches were well visualized. The 4D renderings of the dynamics of the beating *Xenopus* embryo heart are shown in supplementary videos. The systolic and diastolic dynamics of the embryo heart and the flow of the blood cells were clearly observed in their 3D context.

## Conclusion and Discussion

By leveraging the 9.4 MHz speed of the SPML-OCT system, we were able to demonstrate new capabilities in three microscopy applications. First, high-quality OCTA imaging was performed at a rate of 1.3 volumes/s. OCTA is the most widely used functional form of OCT and is used routinely in ophthalmology. As such, improvements in OCTA performance have been in high demand. The increase in OCTA speed demonstrated here can be used to enable wider-field imaging, to reduce motion distortions, and to improve the quality of OCTA images through greater oversampling of each location.

Second, we demonstrated wide dynamic range Doppler OCT imaging of the same rat brain at a rate of 0.53 volumes/s. To enhance the dynamic range of the Doppler OCT to visualize information related to the blood flow speed over wide speed range, it is necessary to acquire multiple Doppler OCT data with a wide range of multiple time intervals. However, performing multiple Doppler OCT imaging with multiple time intervals increases the total imaging time proportionally. With the 9.4 MHz OCT system, eight Doppler OCT volumes with different time intervals ranging from 0.25 to 2.0 ms were acquired for the wide dynamic range Doppler OCT at a rate of 0.53 volumes/s, which is fast enough for most applications. It is worth noting that the phase stability of the system, which is essential for Doppler OCT imaging, is inherently achieved with the SPML laser light source.

Third, we demonstrated video-rate 3D OCT imaging of a beating *Xenopus* embryo heart at a rate of 30 volumes/s. By utilizing the imaging speed of the 9.4 MHz OCT system, we implemented 4D microscopic imaging of the beating *Xenopus* embryo heart at 30 volumes/s. Supplementary videos generated from the 300 volumes acquired continuously over 10 seconds visualized microscopic 3D dynamics of the embryo heart beating at approximately 2 Hz. The relative motion and size of each part of the heart were clearly observed at each time point. Moreover, the flow of the blood cells in the atrium, ventricle, truncus arteriosus, and aortic arches, and the detailed dynamics of the spiral valve and the atrioventricular valve that regulate blood flow were clearly visualized.

Finally, we note a few observations on the current technology platform. It has been well recognized that signal acquisition bandwidth, data transfer rate, and data processing speed should be properly accompanied for the high-speed OCT^34,48^. Related to this, it is important to note that the implementation of the linear-in-wavenumber sweep of the laser in this work has significantly simplified the signal processing required to generate OCT images from the captured signals. As the imaging system becomes faster, beam scanning efficiency becomes increasingly important in defining overall performance. Identifying beam scanning technology with a commensurate speed, which is linear and easily synchronized with the data acquisition, would maximize the utility of the 10 MHz speed provided by the SPML-OCT system. It is noteworthy that the higher-speed SPML lasers become easier to build as their speed increases. This is because higher-speed systems require shorter CFBGs that are easier to fabricate. In this work, we intentionally used a 10-meter-long CFBG to reduce the wavelength sweep speed of the laser to about 10 MHz to secure the depth range. Using one of the fastest digitizers available with a sampling rate of 4 GHz, the depth range was approximately 1.15 mm in tissue with the current 9.4 MHz A-line rate (1,075,270 nm/ms with 88% duty cycle). This places digitizer speed as a clear limiting factor in defining achievable imaging speeds, even for short-range (<3 mm) acquisition. Circular-ranging OCT architectures^48^, which can also leverage the SPML laser but offer acquisition bandwidth advantages through optical-domain compression, can address this challenge for some applications.

## Methods

### Animal preparation

OCTA and Doppler OCT were performed in the rodent brain. All animal experimental procedures were approved by Korea Advanced Institute of Science and Technology (KAIST) Institutional Animal Care and Use Committee (IACUC) guidelines and all efforts were made to minimize the number of animals used and their suffering, in accordance with the Animal Research: Reporting *In Vivo* Experiments (ARRIVE) guidelines. A Sprague Dawley rat was anesthetized with isoflurane during the experiment (3% for induction, 2–2.5% for surgery, and 1.5–2% for imaging, v/v). A cranial window was implanted on the cortex for imaging. After the surgery, the head of the animal was fixed in a custom head holder and imaging was performed^33^. Throughout the surgical and imaging procedures, the body temperature was maintained at 36.5–37.5 °C via a heating pad (Harvard Apparatus 55–7020).

Video-rate 3D OCT imaging of the *Xenopus* embryo heart was also performed. The embryos were obtained from the Korean Xenopus Resource Center for Research. A *Xenopus* embryo at stage 40–50 was anesthetized by ~4% tricaine (3-amino benzoic acid ethyl ester) solution (400 mg tricaine, 97.9 mL DD water, 2.1 mL 1 M Tris (pH 9), adjusted to pH 7 by 1 M HCl) for 5 minutes and inversely mounted on an acrylic block with T-shaped slot for imaging.

### Data processing

**Figure 6:**
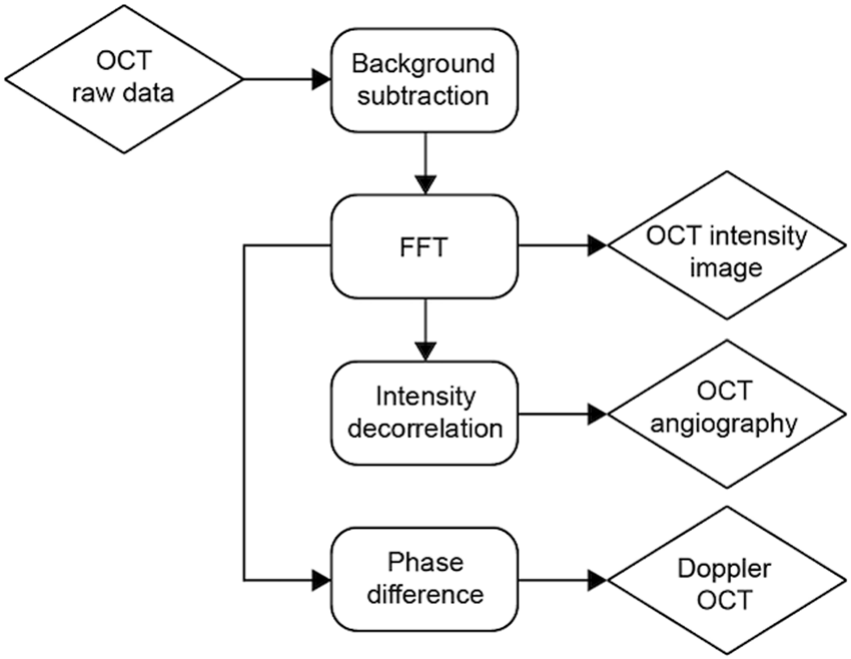
Data processing schema for the SPML-based OCT system.

The data processing schema for the SPML-based OCT system is described in Fig. 6^53^. Since the SPML laser provided a linear-in-wavenumber sweep, a single FFT following a background subtraction generates the OCT A-line. The intensity decorrelations^54^ and phase differences^55^ calculated from a pair of B-scan images acquired at the same location provided OCTA and Doppler OCT cross-sections, respectively.

## Supplementary information

Supplementary Information.

Supplementary Video V1

Supplementary Video V2

Supplementary Video V3
