# Supplementary material for: 9.4 MHz A-line rate optical coherence tomography at 1300 nm using a wavelength-swept laser based on stretched-pulse active mode-locking
Source: Sci Rep. 2020 Jun 9;10:9328. doi: 10.1038/s41598-020-66322-0 (PMC7283258; doi:10.1038/s41598-020-66322-0)
Supplement: Supplementary file 1 — Supplementary Information. [file 41598_2020_66322_MOESM1_ESM.docx]

**SUPPLEMENTARY INFORMATION**

**9.4 MHz A-line rate optical coherence tomography at 1300 nm using a wavelength-swept laser based on stretched-pulse active mode-locking**

Tae Shik Kim^1,2^, JongYoon Joo^1,2^, Inho Shin^1,2^, Paul Shin^1,2^, Woo Jae Kang^1,2^, Benjamin J. Vakoc^3^, Wang-Yuhl Oh^1,2^

^1^Department of Mechanical Engineering, KAIST, Daejeon, Republic of Korea

^2^KI for Health Science and Technology, KAIST, Daejeon, Republic of Korea

^3^Wellman Center for Photomedicine, Massachusetts General Hospital and Harvard Medical School, Boston, MA, USA

**Running title**

9.4 MHz A-line rate optical coherence tomography at 1300 nm

**Corresponding Authors**

**Wang-Yuhl Oh, Ph.D.**

Address: Department of Mechanical Engineering and KI for Health Science and Technology, KAIST, 291 Daehak-ro, Yuseong-gu, Daejeon 34141, Republic of Korea

Tel: 82-42-350-3237, E-mail: woh1@kaist.ac.kr

**Benjamin J. Vakoc, Ph.D.**

Address: Wellman Center for Photomedicine, Massachusetts General Hospital and Harvard Medical School, 40 Blossom Street, Boston, MA, USA

Tel: 1-617-726-0695, E-mail: bvakoc@mgh.harvard.edu

**SUPPLEMENTARY MATERIALS AND METHODS**

**Slow-axis beam scanning**


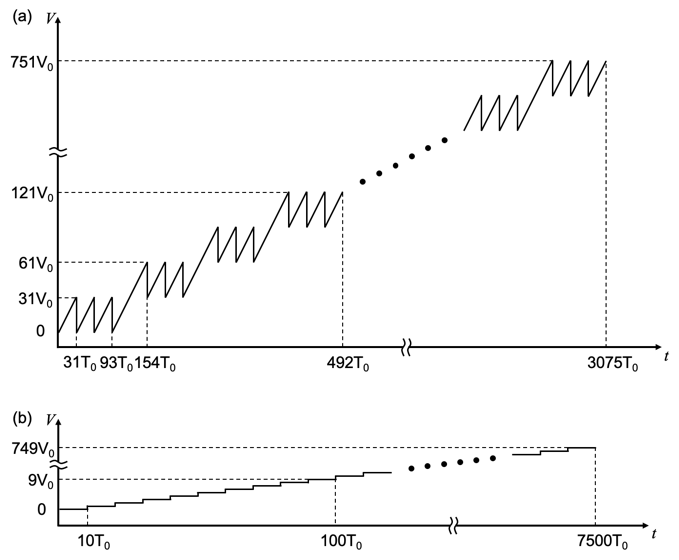
As described in detail in the main article, for the slow-axis beam scanning, we used a stepwise sawtooth scan pattern for the OCTA imaging and a simple stepwise scan pattern for the wide dynamic range Doppler OCT imaging, respectively. Supplementary Fig 1 graphically shows the details of the scan patterns. T_0_ represents a B-scan interval, which is 0.25 ms, and V_0_ represents the slow-axis beam scan voltage corresponding to a successive B-scan spacing. One minor point to note is that we assigned an additional B-scan interval for each flyback in the stepwise sawtooth scan for the OCTA.

**Supplementary Fig 1. Slow-axis beam scanning patterns.**

(a) Stepwise sawtooth scan for a OCTA imaging. (b) Stepwise scan for a wide dynamic range Doppler OCT imaging.

**Wide dynamic range Doppler phase difference**

The wide dynamic range Doppler phase difference Φ was determined by selectively using the Doppler phase difference obtained with each time interval as

$$\Phi[\pi/{2:\pi]}=\varphi_{\tau}[\pi/{2:\pi],}$$

$$\Phi[\pi/{4:\pi/2]}={\frac{1}{2}\varphi}_{2\tau}[\pi/{2:\pi],}$$

$$\Phi[\pi/{6:\pi/4]}={\frac{1}{4}\varphi}_{4\tau}[{2\pi}/{3:\pi],}$$

$$\Phi[\pi/{8:\pi/6]}={\frac{1}{6}\varphi}_{6\tau}[{3\pi}/{4:\pi],}$$

$$\Phi[0:\pi/{8]}={\frac{1}{8}\varphi}_{8\tau}\left[ 0:\pi\right],$$

where τ = 0.25 ms and $\varphi_{T}[A:B]$ is the Doppler phase difference values between A and B acquired with a time interval of T.

**Supplementary Video 1.**

A real-time 3D volume rendered video of a beating Xenopus embryo heart in coronal view. Images were acquired at a rate of 30 volumes per second and displayed at the same rate.

**Supplementary Video 2.**

A real-time 3D volume rendered video of a beating Xenopus embryo heart in axial view. The same data set used for the supplementary video V1 was used to show a volume rendered video in axial view.

**Supplementary Video 3.**

A real-time 3D volume rendered video of a beating Xenopus embryo heart in sagittal view. The same data set used for the supplementary video V1 was used to show a volume rendered video in sagittal view.
